# Designing New Hybrid Antibiotics: Proline-Rich Antimicrobial Peptides Conjugated to the Aminoglycoside Tobramycin

**DOI:** 10.1021/acs.bioconjchem.2c00467

**Published:** 2023-06-28

**Authors:** Stefano Gambato, Ottavia Bellotto, Mario Mardirossian, Adriana Di Stasi, Renato Gennaro, Sabrina Pacor, Andrea Caporale, Federico Berti, Marco Scocchi, Alessandro Tossi

**Affiliations:** † Department of Life Sciences, University of Trieste, Via L. Giorgeri, 5, 34127 Trieste, Italy; ‡ Department of Chemical and Pharmaceutical Sciences, University of Trieste, Via L. Giorgeri, 1, 34127 Trieste, Italy; § CNR, Institute of Crystallography, SS 14 Km 163.5 c/o Area Science Park, Basovizza, 34149 Trieste, Italy; ∥ CIRPeB, Research Centre on Bioactive Peptides “Carlo Pedone”, University of Naples “Federico II”, 80134 Napoli, Italy

## Abstract

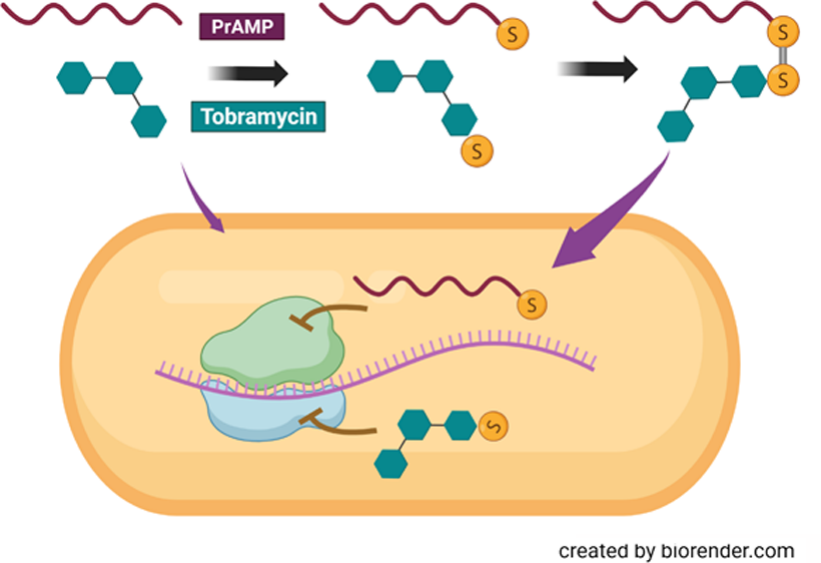

Resistance to aminoglycoside antibiotics is a serious problem, typically arising from inactivating enzymes, reduced uptake, or increased efflux in the important pathogens for which they are used as treatment. Conjugating aminoglycosides to proline-rich antimicrobial peptides (PrAMPs), which also target ribosomes and have a distinct bacterial uptake mechanism, might mutually benefit their individual activities. To this aim we have developed a strategy for noninvasively modifying tobramycin to link it to a Cys residue and through this covalently link it to a Cys-modified PrAMP by formation of a disulfide bond. Reduction of this bridge in the bacterial cytosol should release the individual antimicrobial moieties. We found that the conjugation of tobramycin to the well-characterized N-terminal PrAMP fragment Bac7(1–35) resulted in a potent antimicrobial capable of inactivating not only tobramycin-resistant bacterial strains but also those less susceptible to the PrAMP. To a certain extent, this activity also extends to the shorter and otherwise poorly active fragment Bac7(1–15). Although the mechanism that allows the conjugate to act when its individual components do not is as yet unclear, results are very promising and suggest this may be a way of resensitizing pathogens that have developed resistance to the antibiotic.

## Introduction

Aminoglycosides (AGs) are a group of structurally different amino-modified sugars and represent a clinically important class of antibiotics for their broad spectrum of activity against a broad range of pathogenic bacteria.^1−3^ Their mechanism of action is based on inhibition of bacterial protein synthesis by binding to the 16S rRNA of the bacterial minor ribosomal unit.^4−6^ Despite their efficacy, antibacterial resistance (AMR) to AGs has dramatically increased due to enzymatic inactivation, target modification, reduced uptake and/or drug efflux.^7−9^ AMR represents a serious problem when AGs are used to treat chronic pathologies such as cystic fibrosis (CF),^10^ which is the case with tobramycin.^11^ This antibiotic is part of the standard-of-care in CF patients for dealing with pulmonary infections caused by *Pseudomonas aeruginosa*.^12^ Maintaining the efficacy of tobramycin despite growing bacterial antibiotic resistance would therefore be very important for several infectious diseases.

Antimicrobial peptides (AMPs) are immune effectors that can prevent or combat microbial infections as important components of the innate host defense in multicellular organisms.^13,14^ Most AMPs interact with the microbial surface and act by compromising the integrity of cellular membranes and/or interfering with cell wall synthesis,^15,16^ which is generally reflected in a broad spectrum of antimicrobial activity.^17^ Deshayes et al. conjugated redesigned membrane-active AMPs displaying different sequences, hydrophobicity and helical amphiphilicity, with tobramycin.^18,19^ The purpose of this kind of research was to obtain a unimolecular but multifunctional drug with a broad-spectrum antibiotic activity against antibiotic-resistant strains, using different and synergistic mechanisms to kill them.

In this scenario, Proline-rich AMPs (PrAMPs) may be of interest for conjugate development, since they are characterized by a nonlytic mode of action and are selective for some species of Gram-negative bacteria.^20−22^ Two distinct mechanisms of action have been observed for PrAMPs, both involving protein synthesis: (i) type I PrAMPs allow initiation of protein synthesis but prevent the transition into the elongation phase by hindering the accommodation of tRNA in the A-site,^23−27^ whereas (ii) type II PrAMPs allow initiation and elongation of protein synthesis but hinder termination of translation by trapping release factors in the ribosome.^28,29^

Bac7, a 60-residue, linear peptide isolated from bovine neutrophils, and especially its fragments, are among the best-studied PrAMPs.^20,30^ The derivatives Bac7(1–16) and Bac7(1–35), corresponding to the 16 and 35 N-terminal residues, respectively, showed comparable antimicrobial activity to native, full-length Bac7.^31−33^ On the other hand, removal of only one further C-terminal residue (Arg) from Bac7(1–16) significantly reduces antimicrobial activity.^31,33^ Furthermore, it has been found that to interact with the ribosome and inhibit protein synthesis, Bac7 functional fragments cross the bacterial inner membrane via the SbmA transporter, without permeabilizing it at active concentrations. For this reason, they may be termed bacteria-penetrating peptides (BPPs), as well as AMPs.

We anticipated that the BPP and AMP properties of Bac7 derivatives could be exploited to obtain new bifunctional hybrids by covalently linking them to tobramycin. This would allow targeting bacterial ribosomes via two different inhibitory mechanisms, while likely utilizing different cell internalization mechanisms. To this end we synthesized peptide chimeras derived from both Bac7(1–15) and Bac7(1–35) conjugated with tobramycin, and subjected them to preliminary testing. We linked them by introducing a disulfide bond between tobramycin and PrAMP to develop a conjugate that could be active as such, but with an easily cleavable covalent linker that would also allow release of the active components in the reductive intracellular environment. Should the system work, the aim is to eventually design a system that could do this while avoiding premature release in the extracellular medium or in the blood.^34^

## Results and Discussion

### Synthesis of the Tobramycin-PrAMP Conjugates

**Scheme 1:**
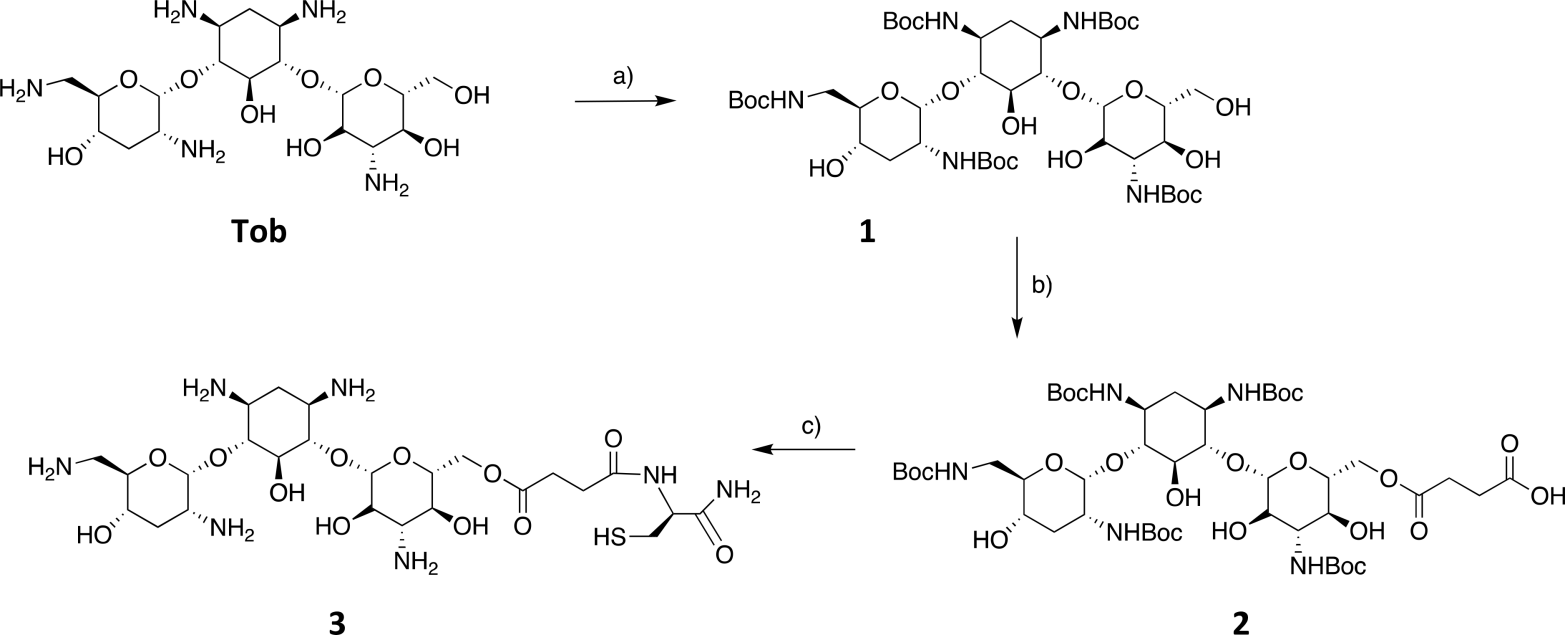
(a) Boc_2_O, H_2_O/DMSO (1:6), 5 h, 60 °C, 80%; (b) Succinic Anhydride (1.5 equiv) and DMAP (5 equiv) in Toluene, Overnight, 85 °C, 60%; (c) Solid Phase Synthesis Followed by TFA/TIPS/DODT/H_2_O (82:5:8:5 v/v), 3 h, rt, > 90% See Experimental Procedures.

The primary hydroxyl group at the 6′ position in tobramycin was chosen as the conjugation point between the peptide and tobramycin (**Tob**) because of its expected higher relative reactivity (compared with the other secondary hydroxyls present) and because functionalization is unlikely to compromise antimicrobial activity.^18^ The primary hydroxyl of tobramycin and other aminoglycosides is in fact not essential for RNA binding.^6,35^ On the other hand, the amino groups are important for antibiotic activity, due to H-bond formation and electrostatic interaction with the ribosome, so they were protected with the *tert-*butyloxycarbonyl (Boc) group to give (Boc)_5_Tob (**1**).^36,37^ The primary 6′-hydroxyl was then selectively functionalized using succinic anhydride to introduce a terminal carboxyl function (**2**)^19^ that allows for coupling with the α-amine of an amidated Cys residue (**3**) (Scheme 1).

The reaction between succinic anhydride and compound **1** was carried out in anhydrous toluene with 4-(dimethylamino)pyridine (DMAP) at 85 °C overnight and was monitored by MS. After purification, the yield of the modified Tobramycin (mTob) (**2**) was ∼60%, in agreement with the literature.^19^ The presence of byproducts was revealed by mass spectroscopic analysis, which indicated the presence of both single (*M* 1068.5) and double hemiacylation (*M* 1168.6). The modification and protection of Tob allowed its direct use in solid-phase peptide synthesis (SPPS), so that compound **2** was coupled to the α-amine of a Cys residue bound to Rink amide resin, adding 2 equiv, with PyBop (0.98 equiv) and DIPEA (2 equiv) as coupling reagents and monitoring the reaction with the Kaiser Test. The product (**3**, mTob-Cys-NH_2_) was then cleaved from the resin using a standard cleavage mixture (Scheme 1c) and the crude material was precipitated with 20 mL of cold *tert*-butyl methyl ether and collected in good crude yield (>90%), indicating efficient coupling under these conditions. ESI-MS (single peak at *M* 670.5) corresponded to compound **3**, and analytical RP-HPLC revealed a sufficient purity for it to be used in subsequent synthetic steps without purification.

**Scheme 2:**
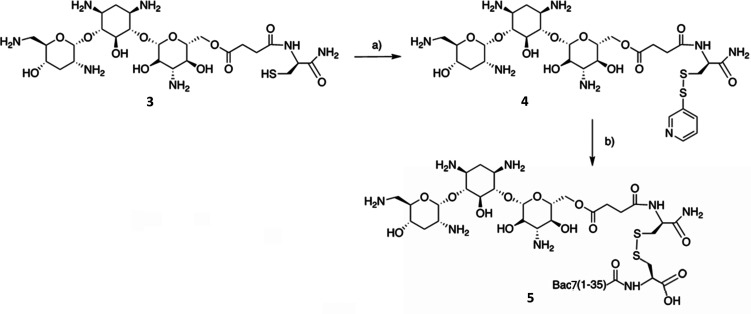
(a) 2,2′-Dithiopyridine, MeOH, 5 h, rt; 70%; (b) Bac7(1-35)[Cys^36^]-OH (**5**), DMSO:H_2_O (1:4), 24 h, rt, 21%. or Bac7(1-15)[Cys^16^]-NH_2_ (Not Reported in Figure) to yield (**6**), 12.5%

For direct coupling of the antibiotic to the selected PrAMPs, the peptides Bac7(1–35) and Bac7(1–15) were extended to contain a C-terminal Cys {Bac7(1–35)[Cys^36^] and Bac7(1–15)[Cys^16^]}. These were synthesized in SPPS using the Trityl and Rink Amide resins respectively, resulting in a peptide amide for the shorter peptide (see Table 1). After purification by RP-HPLC, the correctness of the peptides was confirmed by ESI-MS analysis at *M* 4310.8 and at *M* 2022.3, respectively. A small aliquot of both peptides was alkylated with 2-iodoacetamide and served as controls during biological assays. These are designated as Bac7(1–35)[Cys^36^ALK] and Bac7(1–15)[Cys^16^ALK], respectively (see Table 1). Alkylation was confirmed by ESI-MS analysis (*M* 4368.0 and 2079.5, respectively). To promote the formation of the heterodimer formation during this step, the sulfhydryl group of the Cys residue linked to Tob was preactivated with 2,2′-dithiodipyridine^38^ (Scheme 2).

**Table 1:** Structures of the Modified Tobramycin and Peptides Used in This Work as Well as Their Conjugates, with Calculated and Measured Molecular Masses

Name	Sequence	*M*_calc_^c^	*M*_found_^d^
mTob-Cys-NH_2_	Tobramycin(hemisuccinate)-Cys-NH_2_	670.2	670.5
Bac7(1–15)[Cys^16^]-NH_2_	H-RRIRPRPPRLPRPRPC-NH_2_	2022.5	2022.3
aBac7(1–15)[Cys^16^ALK]-NH_2_	H-RRIRPRPPRLPRPRPC(Alk)-NH_2_	2079.3	2079.5
mTob-Bac7(1–15)[Cys^16^]-NH_2_	mTob-^⌜^C-NH_2_ H-RRIRPRPPRLPRPRP^⌝^C-NH_2_	2690.0	2689.9
Bac7(1–35)[Cys^36^]-OH	H-RRIRPRPPRLPRPRPRPLPFPRPGPRPIPRPLPFPC-OH	4310.3	4310.8
bBac7(1–35)[Cys^36^ALK]-OH	H-RRIRPRPPRLPRPRPRPLPFPRPGPRPIPRPLPFPC(Alk)-OH	4368.3	4368.0
mTob-Bac7(1–35)[Cys^36^]-OH	mTob-^⌜^C-NH_2_ H-RRIRPRPPRLPRPRPRPLPFPRPGPRPIPRPLPFP^⌝^C-OH	4978.7	4978.5

a ALK indicates an acetylated C-terminal Cys thiol, which is also amidated.

b The C-terminal Cys residue is side-chain acetylated but not amidated.

c Calculated using Peptide Mass Calculator (PeptideWeb.com) as the average mass.

d Measured by ESI-MS in positive mode.

This reaction was relatively straightforward, and the ESI-MS spectrum, with a major peak at M = 779.5, confirmed the presence of compound **4** in 70% yield. This was then conjugated to Bac7(1–35)[Cys^36^]-OH or to the shorter Bac7(1–15)[Cys^16^]-NH_2_ in a manner similar to that previously described for linking these peptides to fluorescent dyes.^33^ The reaction was carried out at 1:1 molar ratio of antibiotic and peptide and at a relatively high dilution to limit peptide homodimerization, and was monitored to completion by analytical RP-HPLC. The products were then purified by preparative RP-HPLC and corresponded to the desired heterodimers as confirmed by ESI-MS: *M* 4978.5 and 2689.9 for mTob-Bac7(1–35)[Cys^36^]-OH (**5**) and mTob-Bac7(1–15)[Cys^16^]-NH_2_ (**6**) respectively (see Table 1).

### Antimicrobial Activity

The antimicrobial activity (MIC) of the synthesized conjugates was evaluated against the well-characterized reference strain *E*. *coli* BW25113. Unmodified tobramycin and the Cys-alkylated form of the peptides Bac7(1–35)[Cys^36^ALK]-OH and Bac7(1–15)[Cys^36^ALK]-NH_2_ were used as the free antibiotic and peptide controls, respectively (the use of compound **3** with a free thiol administered in the bacterial growth medium was inappropriate and thiol-alkylated **3** lost activity, see Supporting Information S1). Both mTob-Bac7(1–35)[Cys^36^]-OH and mTob-Bac7(1–15)[Cys^16^]-NH_2_ showed increased antimicrobial activity compared to the corresponding unconjugated peptides. Furthermore, mTob-Bac7(1–35)[Cys^36^]-OH also showed a 4-fold higher activity than the unmodified tobramycin (Table 2) whereas the mTob-Bac7(1–15)[Cys^36^]-OH retained the same activity of the aminoglycoside.

**Table 2:** Minimum Inhibitory Concentrations (MIC) of Tobramycin-Bac7 Conjugates toward Bacterial Reference and Clinical Strains^a^

	MIC^b^ (μM)				
Bacterial strain	Tob	mTob-Bac7(1–35)[Cys^36^]-OH	mTob-Bac7(1–15)[Cys^16^]-NH_2_	Bac7(1–35)[Cys^36^ALK]-OH	Bac7(1–15)[Cys^16^ALK]-NH_2_
*E*. *coli* BW25113	4	1	4	2	8
*E*. *coli* BW25113Δ*sbmA*	16	2	4	8	>32
*A*. *baumannii* ATCC 10606	4	2	2	2	16
*S*. *enteritidis* ATCC 14028	8	1	4	1	32
*P*. *aeruginosa* ATCC 27853	1	1	4	32	>32
*P*. *aeruginosa* PA01	1	1	2	16	>32
*P*. *aeruginosa* PA05	2	1	2	4	>32
*P*. *aeruginosa* PA10	>32	2	4	>32	>32
*P*. *aeruginosa* PA21	>32	2	4	>32	>32
*P*. *aeruginosa* PA22	0.5	1	1	4	>32
*P*. *aeruginosa* PA35	>32	1	4	1	32

a Native tobramycin and Bac7 fragments were used for comparison.

b All experiments were carried out in triplicate and repeated three times, inoculating 2.5 × 10^5^ CFU/mL bacteria in 100% MH broth at 37 °C for 18 h. MIC values were visually evaluated as the lowest concentration at which bacterial growth was inhibited (no turbidity/deposit on bottom of wells).

To rule out the possibility that the antibacterial effects observed with the conjugated compounds were actually due to synergistic effects of tobramycin and PrAMPs released in the medium, we performed a checkerboard assay with Bac7(1–15)[Cys^16^ALK]-NH_2_ and tobramycin, on the *E*. *coli* BW25113Δ*SbmA* strain, where a significant increase in antimicrobial activity was observed on conjugates with respect to the single compounds. The results showed that the MIC of tobramycin against *E*. *coli* did not change with increasing concentration of Bac7(1–15)[Cys^16^ALK]-NH_2_, indicating that there was no synergistic effect between the two compounds (see Supporting Information S2).

Transport across the inner membrane of some Gram-negative species (e.g., *E*. *coli* and *S*. *enteriditis*) via the protein SbmA plays a central role in the mode of action of PrAMPs,^39^ giving them access to bacterial ribosomes. We therefore tested whether the PrAMP component confers this internalization mechanism to **Tob** conjugates. For this purpose, we used a mutant bacterial strain with deletion of the gene encoding the transporter SbmA (*E*. *coli* BW25113Δ*sbmA*). As expected, Bac7(1–15)[Cys^16^ALK]-NH_2_ proved to be inactive in the absence of SbmA and sensitivity to Bac7(1–35)[Cys^36^ALK]-OH also decreased significantly (Table 1). Interestingly, deletion of SbmA also reduced sensitivity toward tobramycin 4-fold. This is consistent with reports that mutation of the *sbmA* gene is common in strains adapted to amikacin, an aminoglycoside related to tobramycin.^40,41^ The role of SbmA in tobramycin’s mode of action therefore deserves further scrutiny. However, it was surprising that both mTob-Bac7(1–35)[Cys^36^]-OH and mTob-Bac7(1–15)[Cys^16^]-NH_2_ essentially retained their antibacterial activity in the absence of the SbmA transporter (Table 2). This would indicate that the conjugate can access an as yet undefined internalization route, possibly through an acquired capacity to perturb the bacterial membrane.

This possibility was investigated by propidium iodide uptake experiments on *E*. *coli* BW25113 cells treated with each of the compounds at their MIC values. The cytofluorimetric profiles (Supporting Information S3) are distinctly different to those caused by the known lytic peptide colistin, suggesting that the **Tob** conjugates do not acquire the capability to permeabilize the bacterial membrane. Nonetheless, they were also different to those of the unconjugated species, which were nonlytic and appeared like untreated controls. The profiles were visibly different with respect to both morphology (in terms of side scatter, SSC) and the PI fluorescence. This suggests that the conjugates have acquired the capacity to somewhat perturb the membrane, while not fully permeabilizing it. Should this be related to an increased cell-penetration capacity, it would help explain their activity toward the *E*. *coli* BW25113Δ*sbmA*.

In any case, an SbmA-independent mode of action indicates an advantage of conjugates over ordinary PrAMPs. First, it may be more difficult for bacteria to develop resistance to these compounds, by mutating this nonessential gene. Second, this appears to be a robust property shared by the shorter and less active 1–15 peptide component, when linked to tobramycin. Third, it would broaden the activity spectrum to bacteria that do not normally carry a gene for this transporter. This is the case with *Pseudomonas aeruginosa*, and indeed most strains of this pathogen are weakly susceptible to PrAMPs.^42^ Until the spread of resistance to this antibiotic, the bacterium was instead efficiently controlled by tobramycin, especially in CF and other chronic diseases.^43,44^ It was therefore of interest to assess if mTob-Bac7(1–15)[Cys^16^]-NH_2_ and mTob-Bac7(1–35)[Cys^36^]-OH were active against a panel of clinically isolated *P*. *aeruginosa* strains, some of which multidrug resistant (e.g., PA10, PA21, and PA-35, see Table 2), and comparing the activity to unconjugated antibiotic and peptide components. The ATCC 27853 strain was included as the reference strain in the screening.

The susceptibility of the different *P*. *aeruginosa* strains to unconjugated tobramycin and the Bac7 fragments was found to be quite variable. As expected, the reference ATCC 27853 strain was quite susceptible to tobramycin, but not to Bac7(1–35)[Cys^36^ALK]-OH, whereas their conjugate was as active as the antibiotic alone. The conjugate with the shorter Bac7(1–15)[Cys^16^ALK]-NH_2_ was somewhat less active than the longer Bac fragment. In the clinical isolates the response was variable. PA01, 05, and 22 were susceptible to the antibiotic (MIC in the 0.5–2 μM range) but also moderately susceptible to Bac7(1–35)[Cys^36^]-OH (MIC in the 4–16 μM range). On the other hand, PA10 and 21 were resistant to both. Curiously, the tobramycin resistant PA35 strain was instead quite susceptible to Bac7(1–35)[Cys^36^]-OH. In any case, it seemed particularly promising that the mTob-Bac7(1–35)[Cys^36^]-OH conjugate was broadly effective against all tested strains (MIC in the 1–2 μM range). Moreover, even the shorter mTob-Bac7(1–15)[Cys^16^]-NH_2_ displayed a significant activity (MIC in the 1–4 μM range). Strains PA10 and PA21 represent the most interesting cases, as they are resistant to both the unconjugated peptides and antibiotic (MIC >32 μM) but become quite sensitive to both the long and short conjugates (MICs respectively of 2 μM and 4 μM).

Overall, the tobramycin-PrAMP conjugates effectively inhibited *E*. *coli* both in the presence and the absence of the SbmA transporter and all seven *P*. *aeruginosa* strains, with MIC values ranging from 1 to 4 μM, indicating that the conjugates are generally as potent and broad-spectrum antimicrobials as the constituent molecules, more effective in a few interesting cases.

In summary, by covalently binding tobramycin to active or inactive Bac7 fragments using versatile synthetic routes, we were able to prepare interesting new antibiotic compounds, and showed that they can overcome the insensitivity of bacterial strains to individual molecules. This favorable effect was observed for reference as well as clinically isolated Gram-negative bacterial strains (*E*. *coli* and *P*. *aeruginosa*) and extends to other Gram-negative species (*A*. *baumanii* and *S*. *enteritidis*). Strategies to enhance antibacterial activity by linking dual-acting antimicrobials, resulting in so-called antimicrobial hybrids, have been widely demonstrated.^45−47^ This could be due to reduced susceptibility to degradation by bacterial enzymes or to efflux systems, and/or to improved cell penetration properties of the hybrid. These hybrids could therefore be used as an alternative to combined treatment with the unlinked antimicrobials. The combined use of unlinked antibiotics has also shown promising results in the treatment of drug-resistant nosocomial bacterial strains,^48−52^ but results in complex dual efficacy, pharmacokinetic, and toxicity profiles. Conjugated hybrid agents would avoid this in drug development.

Further studies are needed to better understand the mechanism of action of our conjugated molecules: specifically, whether they enter the cell and/or act on their intracellular target as a conjugated hybrid or as individual components. Tobramycin and Bac7 fragments were linked by a disulfide bond, so it is expected that the reducing environment in bacterial cells^53,54^ would cause the release of the individual components in the cytosol. Indeed, the disulfide bonds are cellular redox switches. In addition, the Cys residue is linked to tobramycin via a succinic acid bound to the antibiotic by an ester bond, which could also be susceptible to cleavage by bacterial esterases. Therefore, the individual peptides and the modified or unmodified tobramycin could act separately upon entering the bacterial cell, and independently reach their respective target sites in the bacterial ribosome. On the other hand, if conjugate molecules do not separate, they would act as a single, multimodal antibacterial compound that simultaneously binds to and affects the ribosome, with dynamics that do not necessarily overlap with those of the individual components.^6,27^ Future tests using conjugates linked by irreversible bonds and/or the addition of fluorophores to the hybrid molecules may help clarify these mechanistic aspects.

Alternatively, or in addition to this, the advantage conferred by conjugation over the individual components could be due to improved transit to the bacterial surface and/or uptake into the bacterial cells. The conjugated molecules may have gained an increased capacity to cross the external barriers and/or bacterial membrane, allowing both the peptides and tobramycin to reach and inactivate their cytosolic targets more rapidly. This may explain the results obtained with *P*. *aeruginosa* PA10 and PA21 strains, in which only the hybrid molecules inhibited bacterial growth. Our conjugates are therefore promising model systems that point to the usefulness of linking antibiotics to PrAMPs. Clearly, they are unsuitable for systemic use as such, given the reducing properties of plasma,^34^ but point the way toward the development of potentially useful therapeutic agents.

## Experimental Procedures

### Materials

Tobramycin (MW: 467.51 g/mol), reagent grade solvents such as *N,N*-dimethylformamide (DMF), ethyl acetate, dichloromethane (DCM), methanol (MeOH), and acetonitrile (CH_3_CN), as well as *p*-toluenesulfonyl chloride (TsCl), trifluoroacetic acid (TFA), thioanisole, triisopropylsilane (TIS), pyridine, *N,N*-diisopropyl-*N*-ethylamine (DIPEA), triethylamine (TEA), and magnesium sulfate (MgSO_4_), were purchased from Sigma-Aldrich. N-Fmoc-l-amino acids, hydroxybenzotriazole (HOBt), benzotriazol-1-yl-oxytripyrrolidinophophonium hexafluorophosphate (PyBOP) and 1-[bis(dimethylamino)methylene]-1*H*-benzotriazolium 3-oxide tetrafluoroborate 2-(1*H*-benzotriazole-1-yl)-1,1,3,3-tetramethyluronium tetrafluoroborate (TBTU) were purchased from Iris Biotech. 2-Chlorotrityl chloride resin (∼1 mmol/g equiv) and Rink Amide resin (0.35 mmol/g equiv) were obtained from Novabiochem. Müller-Hinton growth medium was from Difco, and 96-well round-bottom microtiter plates were from Sarstedt. Bacterial reference strains were provided by the American Type Culture Collection (ATCC) and by the Deutsche Sammlung von Mikroorganismen and Zellkulturen (DSMZ). Clinically isolated strains were isolated from CF patients as reported previously.^55^

Solid-phase syntheses were performed using an Initiator+ Alstra microwave peptide synthesizer (Biotage). ESI-MS analyses were performed using an Esquire 4000 instrument (Bruker Daltonics). The measured mass was compared to the average mass calculated using the Peptide Mass Calculator (PeptideWeb.com). ^1^H and ^13^C NMR spectra were recorded respectively at 500 and 101 MHz for ^1^H and ^13^C NMR, on a Jeol EX-400 instrument (400 MHz).

#### (Boc)_5_Tob (compound **1**, see Scheme 1)

(Boc)_5_-tobramycin was synthesized as described by Michael et al., 1999.^56^ Briefly, tobramycin (0.25 g, 0.53 mmol, 1 equiv) was dissolved in a DMSO/H_2_O mixture (15 mL, 6:1) and warmed at 60 °C while stirring. Di-*tert*-butyl dicarbonate (1.15 g, 5.5 mmol, 10 equiv) was then added to the mixture. The solution was stirred o.n. at 60 °C, then cooled to RT and 5 mL of 30% aqueous ammonia added to stop the reaction. The white precipitate was collected, washed several times with water and dried to yield 0.404 g (80%). Mass analysis (ESI-MS): *M*_calc_ 967.3 vs *M*_found_ 968.0 for C_50_H_84_N_5_O_21_S). ^1^H NMR: (400 MHz, MeOH-*d*_4_, 25 °C): δ (ppm) = 7.65–7.80 (d, 2H), 7.40–7.50 (d, 2H), 6.87 (s, 1H, NH), 6.45–6.59 (br, 3H, NH), 6.40 (s, 1H, NH), 4.77–5.00 (br, 5H), 4.00–4.18 (br, 3H), 3.12–3.55 (br, 10H), 2.39 (s, 3H), 1.82 (m, 1 H), 1.72 (m, 1H), 1.15–1.50 (m, 47 H).

#### (Boc)_5_Tob-hemisuccinate (mTob) (compound **2**, see Scheme 3)

(Boc)_5_-Tob (0.37 g, 0.38 mmol, 1 equiv) was dissolved in 20 mL anhydrous toluene and treated with succinic anhydride (0.058 g, 0.58 mmol, 1.5 equiv) and 4-(dimethylamino)pyridine (DMAP) (0.23 g, 1.9 mmol, 5 equiv). The solution was heated at 85 °C for 20 h in a paraffin oil bath under argon flux until completion of the reaction (monitored by analytical RP-HPLC and ESI-MS). After cooling to room temperature, 20 mL of dichloromethane and 40 mL of aqueous HCl (pH 2.5) were added to separate organic and aqueous phases. The combined organic layer was washed with brine, dried over Na_2_SO_4_, and the solvent removed to yield 0.171 g (84.6%). Mass analysis (ESI-MS): *M*_calc_ 1068.5 vs *M*_found_ 1068.5 calculated for C_47_H_82_N_5_O_22_). ^1^H NMR (400 MHz, MeOH-*d*_4_, 25 °C): δ (ppm) 5.055.15 (br, 2H), 4.30 (m, 1H), 4.19 (m, 1H), 3.95 (m, 1H), 3.303.90 (br, 13H), 2.55 (m, 2H), 2.65 (m, 2H), 2.13 (m, 1H), 2.01 (m, 1H), 1.65 (m, 1H), 1.45 (m, 46H).

**Scheme 3:**
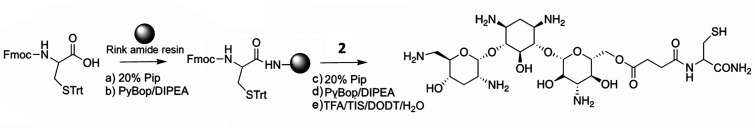
(a) 20% Piperidine in NMP; (b) Fmoc-Cys(Trt)-OH (6 equiv)/PyBop/DIPEA (1:0.98:2) in NMP; (c) 20% Piperidine in NMP; (d) mTob (2 equiv)/PyBop/DIPEA (1:0.98:2) in DMF; (e) TFA/TIS/DODT/H2O (82:5:8:5 v/v)

#### Tobramycin(hemisuccinate)-Cys (compound **3**, see Scheme 1)

This was synthesized in the solid-phase using an automated synthesizer. After deprotection of Fmoc-Rink amide resin (0.266 g, 0.56 mmol/g) with 20% piperidine in NMP, Fmoc-Cys(Trt)-OH/PyBop/DIPEA (1:0.98:2) in NMP were added to the resin with a 6 equiv excess of amino acid. The coupling yield was determined as 0.35 mmol/g by measuring the absorbance of *N*-(9-fluorenylmethyl)piperidine complex at 301 nm after treatment with piperidine. Compound **2** (0.17 g, 0.16 mmol), PyBop (0.082 g, 0.157 mmol, 0.98 equiv), and 56 μL of DIPEA (0.314 mmol, 2 equiv) were dissolved in 1.5 mL of DMF and added to the H-Cys-Rink-amide resin. The reaction was monitored to completion with the Kaiser test after 3 h. The resin was then washed, dried under an N_2_ flux, and the product cleaved from the resin by treating with 1 mL of TFA/TIS/DODT/H_2_O (82:5:8:5 v/v) for 3 h, and precipitated with cold *tert*-butyl methyl ether to yield 49 mg (>90% yield). It was sufficiently pure for use in subsequent reactions without purification. Mass analysis (ESI-MS): *M*_calc_ 670.2 vs *M*_found_ 670.5 calculated for C_47_H_82_N_5_O_22_).

#### Synthesis of Tobramycin(hemisuccinate)-Cys-thiopyridine (compound **4**, see Scheme 2)

Compound **3** (0.049 g, 0.073 mmol) was suspended in 3 mL of methanol and then 2,2′-dithiodipyridine (0.016 g, 0.073 mmol) was added. The reaction was stirred for 5 h at RT and was monitored by RP-HPLC and ESI-MS. The crude product was precipitated with 20 mL cold *tert*-butyl methyl ether, collected, and dried under N_2_ flux. The yield of dried crude product was 40 mg (70.2%). Mass analysis (ESI-MS): *M*_calc_ 779.4, vs *M*_found_ 779.3 for C_47_H_82_N_5_O_22_).

#### Synthesis of Bac7(1–35)[Cys^36^]-OH and Bac7(1–15)[Cys^16^]-NH_2_

Peptides were synthesized using an automated microwave peptide synthesizer. SPPS of Bac7(1–35)[Cys^36^]-OH and Bac7(1–15)[Cys^36^]-NH_2_ were carried out respectively on Fmoc-Cys(Trt)-2-chlorotrityl chloride resin (0.15 mmol/g equiv) and NovaPEG Rink Amide Resin LL (0.16 mmol/g equiv). Coupling was typically carried out with a 5-fold excess of Fmoc-amino acid/HOBt/TBTU/DIPEA (1:1:0.98:2 v/v) in NMP. The coupling temperature was 75 °C, when using the Rink Amide resin but kept at 50 °C with the 2-chlorotrityl chloride resin to prevent premature detachment. Cleavage was performed with a TFA/TIS/DODT/H_2_O (82:5:8:5 v/v) mixture for 3 h. The products were precipitated with cold *tert*-butyl methyl ether, collected, and dried overnight under vacuum. Analysis of the crude peptides by RP-HPLC showed them to be relatively pure so they were used in the next steps without further purification. Analytical and preparative RP-HPLC were respectively carried out using Waters Symmetry C18, 100 Å, 3.5 μm, 4.6 × 75 mm and Phenomenex Jupiter C18, 300 Å, 5 μm, 10 × 100 mm columns. ESI-MS: Bac7(1–35)[Cys^36^]-OH *M*_calc_ 4310.3 vs *M*_found_ 4310.8; Bac7(1–15)[Cys^16^]-NH_2_*M*_calc_ 2022.5 vs *M*_found_ 2022.3.

### Peptide Alkylation with Iodoacetamide

A small aliquot of each peptide was alkylated using 2-iodoacetamide. Two mg of Bac7(1–35)[Cys^36^]-OH (or Bac7(1–15)[Cys^16^]-NH_2_) were dissolved in 300 μL of 10 mM HCl, and divided into five aliquots of 60 μL. The reaction was performed in the dark and under nitrogen. Twenty-five μL of iodoacetamide (Iaa) (stock solution 2 mM Iaa in ethanol) was diluted in 125 mL of 0.5 M Tris-acetate and 2 mM EDTA at pH 8. Then 5 mL of 0.1 mM ascorbic acid (Sigma) was added at room temperature and the reaction was maintained under gentle agitation for 30 min. Another four aliquots were added every 30 min under the same conditions, and the fourth one was immediately followed by 8 mL of 10 mM Iaa dissolved in Tris, pH 8, and 2 mL of 1 mM ascorbic acid to scavenge traces of iodine. The reaction mixture was then diluted with 0.05% trifluoroacetic acid in water to a final pH of 2.5 and the peptides were directly purified by RP-HPLC. Purity was confirmed by analytical RP-HPLC and ESI-MS (Bac7(1–15)[Cys^16^ALK]-NH_2_, yield 56%, *M*_calc_ 2079.3 vs *M*_found_ 2079.5; Bac7(1–35)[Cys^36^ALK]-OH, 66%, *M*_calc_ 4368.3 vs *M*_found_ 4368.0.

#### Synthesis of mTob-Bac7(1–35)[Cys^36^]-OH and mTob-Bac7(1–15)[Cys^16^]-NH_2_, (compounds **5** and **6**, see Scheme 2)

Compound **4** (2 mg, 0.0025 mmol) was suspended in 4 mL of 20% v/v DMSO in H_2_O (pH 5). Bac7(1–35)[Cys^36^] (10.7 mg, 0.0025 mmol) dissolved in 1 mL of H_2_O was slowly added dropwise. The mixture was then stirred for 24 h at RT and the reaction was monitored by RP-HPLC and ESI-MS until its completion. The conjugate was purified by RP-HPLC with a Phenomenex Jupiter C18, 300 Å, 5 μm, 10 × 100 mm column using a gradient 5–35% CH_3_CN in 50 min, with a 2 mL/min flow rate. The collected peak was confirmed by ESI-MS to correspond to the correct heterodimer which was collected with satisfactory yield (2.6 mg, 21%) and good purity. ESI-MS: *M*_calc_ 4978.7 vs *M*_found_ 4978.5. The lyophilized conjugate was accurately weighed and dissolved in slightly acidic water (pH 4.8), to prevent the risk of the solution becoming basic, a condition in which the hemisuccinate linker is unstable. The peptide concentration was determined from both the weight and spectrophotometrically by the Waddle method. The same procedure was used for the synthesis of mTob-Bac7(1–15)[Cys^16^]-NH_2_ using 3 mg, 0.004 mmol of compound **4** and 7.8 mg, 0.004 mmol Bac7(1–15)[Cys^16^]-NH_2_. The product was confirmed by analytical RP-HPLC and the yield after purification was 1.5 mg (12.5%). ESI-MS *M*_calc_ 2690.0 vs *M*_found_ 2689.9.

### Antimicrobial Activity

Minimum inhibitor concentrations (MIC) were determined as previously reported.^57^ Briefly, bacteria were grown overnight (∼18 h) at 37 °C under vigorous shaking (140 rpm) in Müller-Hinton broth (MHB). The day after, 300 μL of bacterial suspension were diluted in 10 mL of fresh MHB and grown at 37 °C until an optical density (A_600_) of ≈0.3 was reached. Meanwhile, the compounds to be tested were serially 2-fold diluted in MHB to a final volume of 50 μL in the wells of a round-bottom microtiter plate. 50 μL of a suspension of 5 × 10^5^ bacteria/mL in MHB was then added to the wells containing the compounds, halving therefore the final concentration of both microorganisms and antimicrobial compounds. The plate was sealed with Parafilm to reduce evaporation and incubated overnight at 37 °C (approximately 18 h). The day after, the plate was visually inspected and the MIC calculated as the minimum concentration of compound resulting in no visible bacterial growth in the wells.

## Supporting Information Available

The rationale for selection of appropriate controls for antimicrobial assays (S1), Checkerboard assay for evaluation of synergistic effects among compounds (S2), Propidium iodide uptake assay for evaluation of bacterial membrane integrity (S3) (PDF)
