# Supplementary material for: Designing New Hybrid Antibiotics: Proline-Rich Antimicrobial Peptides Conjugated to the Aminoglycoside Tobramycin
Source: Bioconjug Chem. 2023 Jun 28;34(7):1212–20. doi: 10.1021/acs.bioconjchem.2c00467 (PMC10360068; doi:10.1021/acs.bioconjchem.2c00467)
Supplement: Supplementary file 1 — bc2c00467_si_001.pdf [file bc2c00467_si_001.pdf]

Supporting information

Designing New Hybrid Antibiotics: Proline-Rich Antimicrobial Peptides Conjugated to the Aminoglycoside Tobramycin

Stefano Gambato,[1,a] Ottavia Bellotto,[2] Mario Mardirossian[1], Adriana Di Stasi[1], Renato Gennaro[1], Sabrina Pacor[1], Andrea Caporale[3,4], Federico Berti[2], Marco Scocchi[1,]* and Alessandro Tossi[1,]*

1. Department of Life Sciences, University of Trieste, 34127 Trieste, Italy
2. Department of Chemical and Pharmaceutical Sciences, University of Trieste, 34127 Trieste, Italy
3. CNR, Institute of Crystallography, 34149 Trieste, Italy
4. CIRPeB, Research Centre on Bioactive Peptides "Carlo Pedone", University of Naples, "Federico II", 80134 Napoli, Italy
a: Present address: SERICHIM S.r.l., 33050 Torviscosa, Udine, Italy
**Corresponding Authors: mscocchi@units.it and atossi@units.it*

Supporting information S1: Selection of appropriate controls for antimicrobial assays

Bac7(1-35)[Cys^{36}]-OH and Bac7(1-15)[Cys^{16}]-NH_2, both having cysteine thiols alkylated using iodoacetamide, were used as a control for the activity of the PrAMPs on their own. On the other hand, the molecular species to use as control for the antibiotic component was somewhat of a quandary. For conjugation with the peptide moiety, the antibiotic was in fact modified by first adding succinic acid and then coupling this to a cysteine. The peptide-antibiotic conjugate could then enter the bacterium and target ribosomes as such, or the antibiotic could be released by reduction of the disulphide bond (therefore acting as cysteine-tobramycin) or by cleavage of the ester bond (therefore acting as free tobramycin).

We considered at first using compound **3** as control for antimicrobial activity of tobramycin, but we felt this was probably not appropriate, since it would not simulate what happens on disulphide bridge reduction in the bacterial cytoplasm. If administered in the bacterial growth medium, during antimicrobial assays, the antibiotic with the free sulfhydryl would likely dimerize or covalently link to medium components, and this would confer quite different properties with respect to the monomer. We therefore considered using a thiol-protected form of compound **3** (mTob-[CysALK]-NH_2) but felt that the mode of action and/or the uptake could be markedly affected by alkylation. Preliminary assays with alkylated compound **3** performed against *E. coli* ATCC 25922 did indeed seem to support this hypothesis, as its MIC was higher than the expected (16 µM), when compared to that of free tobramycin (4 µM). For these combined reasons, we ended up deciding that the unmodified tobramycin and not compound **3** (alkylated or not) was the most appropriate control.

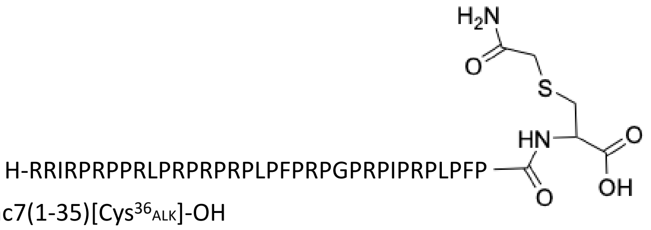

Bac7(1-35)[Cys^{36}_{ALK}]-OH

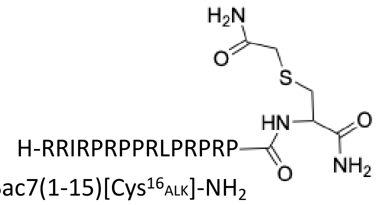

Bac7(1-15)[Cys^{16}_{ALK}]-NH_2

Fig. S1: Bac7(1-35)[Cys^{36}]-OH and Bac7(1-15)[Cys^{16}]-NH_2, with cysteine thiols alkylated by iodoacetamide.

Supporting information S2: Checkerboard assay

The activity of the peptide Bac7(1-15)[Cys^{16}ALK]-NH_2 in combination with tobramycin against the *E. coli* BW25113Δ*sbmA* strain was evaluated using the checkerboard technique. Briefly, both the peptide Bac7(1-15)[Cys^{16}ALK]-NH_2 and Tobramycin were serially two-fold diluted to a final volume of 33 µL at a concentration which is 3 fold higher than the one to be tested in the assay as final concentration. Peptides and tobramycin at different concentrations were then mixed in equal proportions in the wells of a 96-well round-bottom microtiter plate to a final volume of 66 µL. A mid-log bacterial culture of *E. coli* BW25113Δ*sbmA* was diluted to a bacterial load of 7.5×10^5 CFU/mL and 33 µL of this suspension were then added to each well of the plate (except for the sterility control wells in MHB), reaching the final desired concentration of peptides and bacteria. The plate was then sealed with parafilm to reduce evaporation and incubated at 37 °C for 18 hours.

The combined use of Bac7(1-15)[Cys^{16}ALK]-NH_2 and Tobramycin at concentrations corresponding to the MIC reported for the mTob-Bac7(1-15)[Cys^{16}]-NH_2 , i.e. 4 µM (see Table 2), did not impair bacterial growth. There was no indication of improvement in the antimicrobial activity of Tobramycin due to the contemporary use of Bac7(1-15)[Cys^{16}ALK]-NH_2 up to its highest concentration of 16 µM, when bacterial growth was inhibited by the combined use of the peptide with 2 µM of antibiotic (Figure S2). The assay therefore suggests that there is no marked synergic activity between the free Bac7(1-15)[Cys^{16}ALK]-NH_2 and Tobramycin, indicating rather the possibility of an additive effect [1].

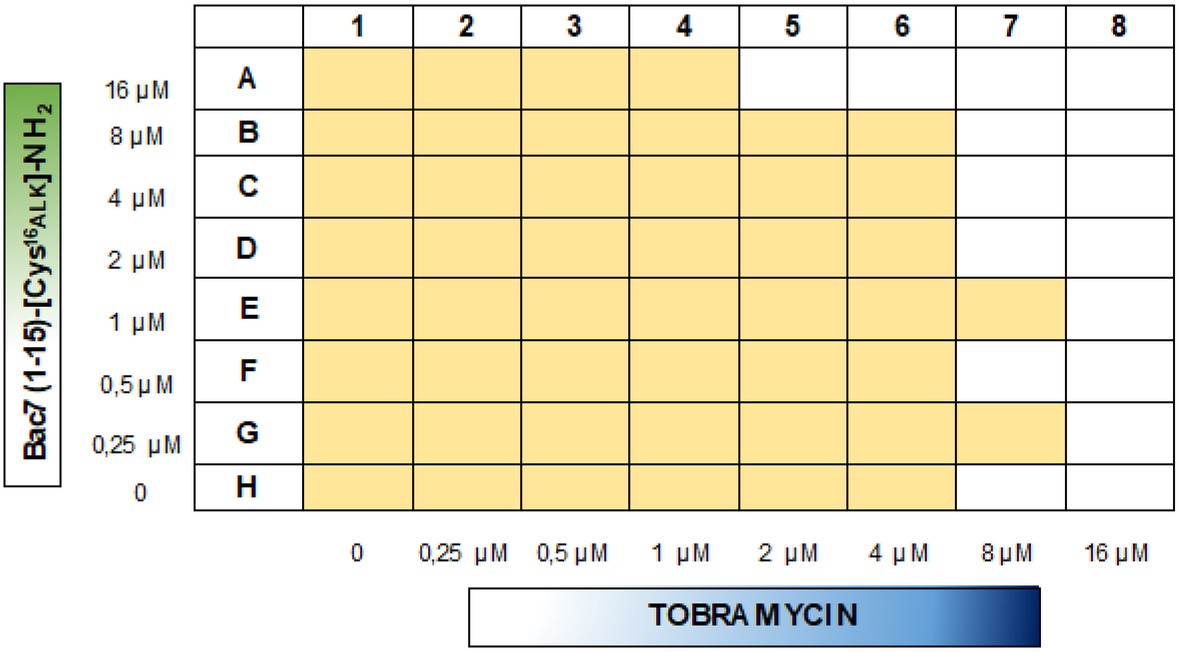

Fig. S2: The plate set-up procedure requires that columns 1 to 8 contain serial twofold dilutions of tobramycin (ranging from 0.25 to 16 µM), while rows A to G contain serial twofold dilutions of Bac7(1-15)[Cys^{16}ALK]-NH_2 (ranging from 0.25 to 16 µM). Row H contains serial dilutions of tobramycin alone, while column 1 contains serial dilutions of Bac7(1-15)[Cys^{16}ALK]-NH_2 alone. In these columns, the MIC value for each compound can be determined individually. In the figure, the yellow color represents the wells where there was visible bacterial growth, while a white color indicates no significant growth. No combination of the different concentrations of Bac7(1-15)[Cys^{16}ALK]-NH_2 resulted in a synergistic effect (Fractional index concetration, FIC < 0.5).

Supporting information S3: Propidium iodide uptake assay

Flow cytometric analyses of membrane permeabilization were performed as described previously [2,3]. Briefly, mid-exponential cultures of *E. coli* BW25113 (2.5×10^5 CFU/ml) in 100% MHB were treated with peptide or antibiotic concentrations corresponding to MIC values for 30 min at 37°C. Propidium iodide (PI) was added immediately prior to flow cytometry acquisition, to final concentration of 10 µg/ml. This was gated in order to avoid cell clumping; the red fluorescence from PI emitting, damaged bacteria, was detected at 610 nm and shown as dot plots (Side Scatter *vs* PI fluorescence). Single parameter histograms for PI fluorescence are also shown for the negative control (untreated sample) and for the known membranolytic peptide antibiotic colistin, used as positive control. Measurements were carried out using an AttuneTM NxT (ThermoFisher), equipped with a Blue laser (488 nm, 50 mW) and standard optical bench configuration. After acquisition of at least 10000 events for each run, data were stored as list mode files and analyzed using FCS Express V7 (De Novo Software).

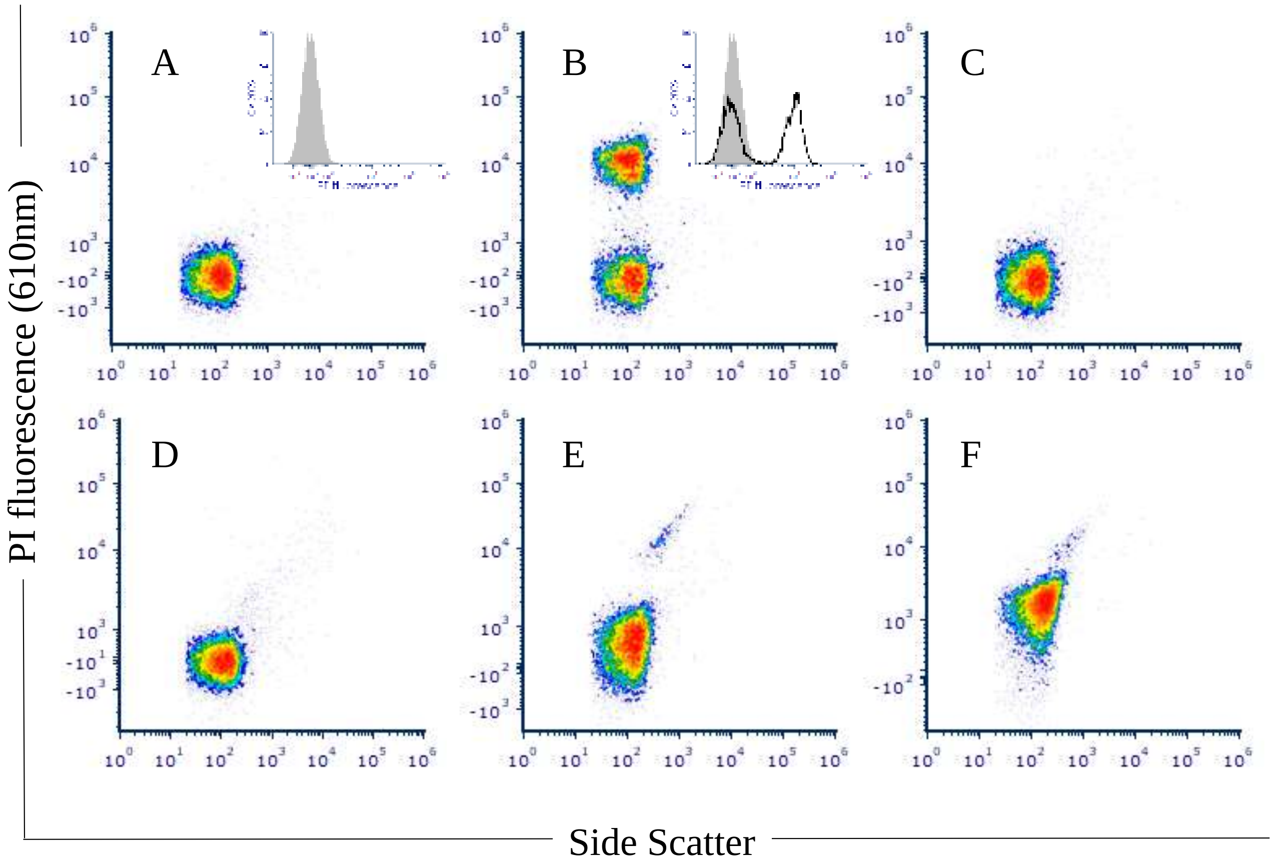

Fig. S3: Effect of treatment with unconjugated and conjugated Tobramycin and PrAMPs on PI uptake. Flow cytometric analysis of *E. coli* BW25113 treated for 30 min with Tobramycin (C), Bac7(1-35)[Cys^{36}ALK]-OH (D) or the conjugates mTob-Bac7(1-15)[Cys^{16}]-NH$_2$ (E) and mTob-Bac7(1-35)[Cys^{36}]-OH (F). Controls are shown respectively in (A) Untreated and (B) Colistin-permeabilized bacteria. Dot-plots showing Side Scatter (X)/PI red fluorescence (Y) signals are representative of repeated experiments. Data in-set in (A) and (B) display the number of events distributed according to the PI-fluorescence for the negative and positive controls. The overlay of bacterial populations of the untreated control (gray shaded) and colistin-treated sample (empty black line) is shown in (B).

Supplementary references

1) Orhan G.; Bayram A.; Zer Y.; Balci I. Synergy Tests by E Test and Checkerboard Methods of Antimicrobial Combinations against *Brucella melitensis*. *J. Clin. Microbiol.* **2005**, 140–143.
2) Pacor, S.; Benincasa, M.; Musso, M. V.; Krce, L.; Aviani, I.; Pallavicini, A.; Scocchi, M.; Gerdol, M.; Mardirossian, M. The Proline-Rich Myticalins from *Mytilus galloprovincialis* Display a Membrane-Permeabilizing Antimicrobial Mode of Action. *Peptides* **2021**, 143, 170594.
3) Pacor, S.; Guida, F.; Xhindoli, D.; Benincasa, M.; Gennaro, R.; Tossi, A. Effect of Targeted Minimal Sequence Variations on the Structure and Biological Activities of the Human Cathelicidin LL-37. *Peptide Sci.* **2018**, 110 (5), e24087.
